# Assessment of fertility protection and ovarian reserve with GnRH antagonist in rats undergoing chemotherapy with cyclophosphamide

**DOI:** 10.1186/1477-7827-8-51

**Published:** 2010-05-18

**Authors:** Claudia NCD Lemos, Fernando M Reis, Guilherme N Pena, Laila C Silveira, Aroldo F Camargos

**Affiliations:** 1 Laboratório de Reprodução Humana Prof Aroldo Fernando Camargos, Hospital das Clínicas, Universidade Federal Minas Gerais, Minas Gerais, Brazil

## Abstract

**Background:**

Reproductive function following chemotherapy is of increasing importance given that survival rates are improving. We assessed whether a gonadotropin-releasing hormone antagonist (GnRHant; cetrorelix) could promote ovarian protection against damage due to chemotherapy.

**Methods:**

Forty-two female Wistar rats were used in this study. Animals were divided into four groups: group I (n = 9) received placebo twice; group II (n = 12) received placebo + cyclophosphamide (CPA); group III (n = 12) received GnRHant + CPA; and group IV (n = 9) received GnRHant + placebo. After medication, the estrous cycle was studied through vaginal smears. Rats were mated, pregnancy was documented and the number of live pups evaluated. Afterwards, rat ovaries were removed and prepared for histological studies. The ovarian cross-sectional area was measured and follicles were counted.

**Results:**

Cyclic changes in vaginal smears were observed in all but one animal after treatment, but group II had a significantly lower rate of animals with proestrus or estrus (p < 0.01). The offspring was markedly reduced by CPA treatment (group II, 3.00 +/- 1.33 pups vs. group I, 11.44 +/- 0.78 pups, p < 0.01) and this effect was partly reversed by pre-treatment with GnRHant (group III, 7.00 +/- 1.31 pups). The ovarian cross-sectional area was not significantly different between groups, neither was the number of individual follicle types. However, rats in Group IV had a higher total number of ovarian follicles than those in the control group (17.1 +/- 1.22 vs. 10.9 +/- 0.70, p < 0.05).

**Conclusion:**

The use of a GnRHant before CPA chemotherapy provided protection of fertility.

## Background

Current advances in cancer treatment have substantially increased the survival of reproductive age women with cancer [1-4] and, as survival rates improve, the quality of life of these patients deserves more attention. The loss of reproductive function is one of the most important adverse effects of chemotherapy. Cancer treatment with DNA alkylating agents, such as cyclophosphamide (CPA), can lead to impaired fertility or ovarian failure, resulting in premature menopause [5]. CPA is widely used in the treatment of malignant neoplasms and some auto-immune diseases, including rheumatoid arthritis and systemic lupus erythematosus.

New strategies have been developed to prevent the adverse effects of chemotherapy on ovarian function. Dividing cells are known to be more sensitive to the cytotoxic effects of the alkylating agents than are cells at rest. It has been suggested that inhibition of the pituitary-gonadal axis would reduce the rate of oogenesis and thereby render the germinal epithelium less susceptible to the effects of chemotherapy [1]. For this purpose, the use of gonadotropin-releasing hormone (GnRH) agonists prior to chemotherapy is currently under investigation. Ozcelik *et al*. [6] and Tan *et al*. [7] showed a protective effect of a GnRH agonist on chemotherapy induced ovarian gonadoxicity in mice and rats, respectively. The same protective effect was found in a randomized clinical trial [8] and a meta-analysis [9]. At least four ongoing randomized clinical trials are addressing the issue of ovarian preservation with GnRH agonists [10-13].

GnRH agonists have an initial stimulatory effect on the gonads, thus delaying ovarian suppression, which is expected to occur after a 14-day period. This waiting time is crucial when the treatment of cancer is proposed [14]. In contrast, GnRH antagonists cause immediate ovarian suppression by competitively blocking GnRH receptors in the pituitary. With their immediate onset of action, GnRH antagonists may be suitable for use immediately before initiation of chemotherapy. However, there are no animal studies to date that have assessed the role of a GnRH antagonist on fertility protection during chemotherapy. We also have not found any published clinical trial using GnRH antagonist with this objective.

Thus, the aim of the present study was to investigate whether the administration of a GnRH antagonist prior to the administration of CPA in female rats would prevent ovarian damage and promote fertility preservation.

## Methods

This controlled experimental study was carried out at the animal research unit of the Faculdade de Medicina, Universidade Federal Minas Gerais (UFMG), at Belo Horizonte, Brazil. The study followed the international ethical guidelines and was approved by UFMG Ethics Committee on Animal Experimentation.

### Animals

A total of 42 female Wistar rats 60 days old with a mean weight of 350 g were used. Animals were obtained from the animal research facility at the Instituto de Ciências Biológicas of UFMG. Prior to the study, the estrous cycle was confirmed by vaginal cytology, and only those rats with an estrous cycle of 4-7 days were included [15].

Rats were kept in Alesco plastic cages, model ALE.MIL. 01. 05, measuring 70 × 40 × 20 cm. No more than five animals were kept together in each cage. Room temperature was maintained at 24 ± 2°C, with a 12-hour light cycle and adequate air supply. Water and rat food pellets were available *ad libitum*.

### Medication

#### GnRH antagonist (cetrorelix)

A diluted solution of 0.1 mg/kg of cetrorelix acetate in distilled water and 5% mannitol was administered intraperitoneally 1 hour before the CPA injection [16,17]. Previous studies had shown that the maximum serum concentration of cetrorelix is reached after 2 hours. This dose is known to inhibit the luteinizing hormone surge in about 1 hour.

#### Cyclophosphamide

A diluted solution of 6 mg/kg of CPA in 0.9% sodium chloride (saline), resulting in a 1 mg/mL dose, was administered intraperitoneally. This dose is reported to have a mortality rate of 24% and to induce a decrease in fertility in about 70% of the animals [18,19].

#### Placebo

Saline solution was used as placebo.

### Drug administration schedule

Drugs were injected aseptically via the peritoneum, on the inferior abdominal area next to the right hind leg, in three cycles of five consecutive days followed by a 2-day drug-free interval. Drugs were administered in the mornings and the treatment course took 3 weeks to complete, according to the following schedule:

• Group I (control; n = 9). Initial treatment: placebo injection. After 1 hour, another placebo injection was given.

• Group II (n = 12). Initial treatment: placebo injection. After 1 hour, a CPA injection was given.

• Group III (n = 12). Initial treatment: GnRH antagonist (cetrorelix) injection. After 1 hour, a CPA injection was given.

• Group IV (n = 9). Initial treatment: GnRH antagonist (cetrorelix) injection. After 1 hour, a placebo injection was given.

Doses were adjusted weekly based on the weight of the animal.

### Evaluation of fertility

The day after the final treatment, the rats were submitted to a new 8-day estrous cycle study in order to confirm and document fertility status. They were then allowed to mate with adult male Wistar rats. Mating was confirmed by the presence of spermatozoa on microscopic analysis of vaginal wash. Confirmation was considered as the first day of pregnancy. After 21 days, the offspring were counted.

After delivery, the rats were killed with a peritoneal injection of 35 mg sodium thiopental and underwent bilateral oophorectomy. Each ovary was entirely cut into serial longitudinal sections and follicle count was performed at four sections distant from each other by approximately 200 μm. This method was chosen considering (i) that other published studies with similar design evaluated a few random ovarian sections [6,20]; (ii) there is a strong correlation between serial and random section counting for the number of small follicles [20]; and (iii) keeping a regular distance between counted sections would avoid duplicate counting of large follicles. The sections were stained with hematoxylin and eosin and the total number of each type of follicle (primordial, primary, preantral and antral) and corpora lutea was computed for every section, then used to calculate the average number of follicles per section for every animal. The largest ovarian section from each rat was selected and its area was calculated using Image Pro-plus version 6.0 software (Media Cybernetics, Bethesda, MD, USA).

### Statistical analysis

Results were analyzed with Prism version 4.0 software (GraphPad, La Jolla, CA, USA). Data were normally distributed and therefore expressed as mean ± standard error (SE). Differences between groups were tested by one-way analysis of variance (ANOVA). If a significant overall difference was found, the post hoc Newman-Keuls test was computed for multiple comparisons. Qualitative data were evaluated by likelihood ratio chi-square test. A value of p < 0.05 was considered statistically significant.

## Results

Following treatment, two rats died in Group II and none in the remaining groups, leaving for analysis n = 9, n = 10, n = 12 and n = 9 animals in groups I, II, III and IV, respectively.

**Figure 1:**
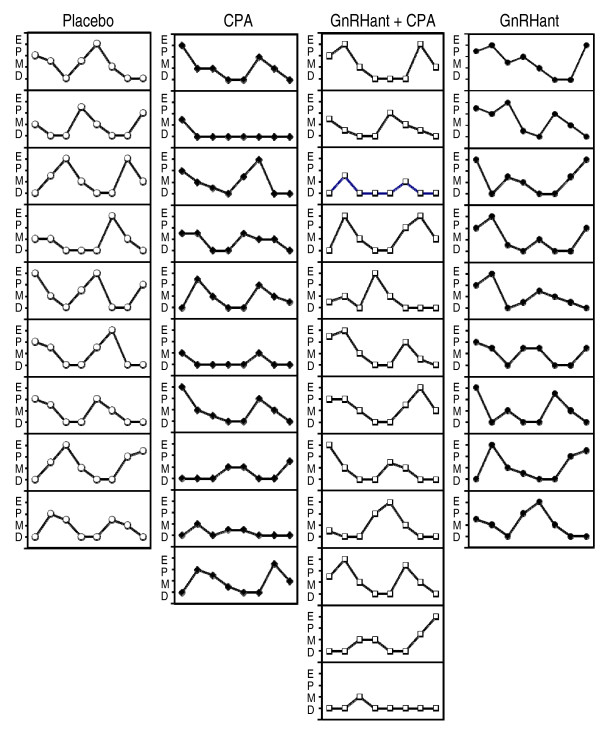
**Individual plots showing the estrous cycles recorded during 8 days, shortly after the conclusion of chemotherapy course with placebo, cyclophosphamide (CPA), cetrorelix (GnRHant) plus CPA, and GnRHant**. The vertical axes represent the phases of estrous cycle (D = diestrus; M = metaestrus; P = proestrus; E = estrus) and the horizontal axes represent the time (days).

Eight-day samples of estrous cycles following treatments are shown in Figure 1. Cyclic changes in vaginal cytology were observed after treatment in all but two animals (rat 2 from Group II and rat 12 from group III), which entered permanent diestrus. However, CPA induced a significantly lower rate of cycles containing at least one proestrus and/or estrus, and this effect of CPA was reversed by pre-treatment with GnRHant (chi-square = 12.42, p < 0.01). Those cycles were detected in 9/9 rats from Group I, 5/10 rats from group II, 10/12 rats from group III and 9/9 rats from group IV (Table 1).

**Table 1:** Summary of estrous cycles recorded during 8 days, shortly after the conclusion of chemotherapy course.

	Group I (placebo)	Group II (CPA)	Group III (GnRHant + CPA)	Group IV (GnRHant)	Chi-Square (p value)
Number of surviving rats per group	9	10	12	9	-
Number of rats with a cyclic pattern (%)	9 (100)	9 (90)	11 (92)	9 (100)	2.50 (p = 0.48)
Number of rats with estrus/proestrus (%)	9 (100)	5 (50)	10 (83)	9 (100)	12.42 (p < 0.01)

CPA, cyclophosphamide; GnRHant, gonadotropin-releasing hormone antagonist (cetrorelix).

**Figure 2:**
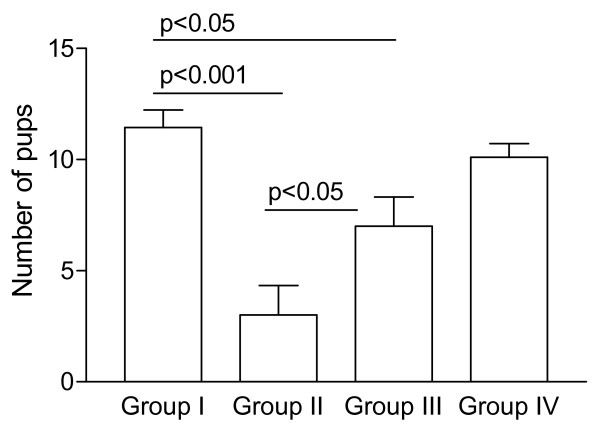
**Number of live pups per litter in rats treated with placebo (Group I), cyclophosphamide (Group II), cetrorelix plus cyclophosphamide (Group III), and cetrorelix (Group IV)**. Data are expressed as means ± SE and the group means were compared by the Newman-Keuls test.

Twenty-one days after mating, the offspring from each animal was counted. Figure 2 shows a comparison of the mean number of live pups per litter in each group. The offspring was markedly reduced by CPA treatment (Group II, 3.00 ± 1.33 pups vs. Group I, 11.44 ± 0.78 pups, p < 0.01) and this effect was partly reversed by pre-treatment with GnRHant (Group III, 7.00 ± 1.31 pups). The group that received only GnRHant (Group IV, 10.11 ± 0.61 pups) did not differ from the placebo-treated controls. There was no case of perinatal death and the pups were apparently of normal size and morphology.

The mean ovarian cross-sectional area ranged from 4.30 to 5.45 mm^2 ^and was not significantly different between groups (Table 2). The average numbers of primordial and primary follicles, preantral follicles, antral follicles and corpora lutea were not altered by treatment with CPA or CPA + GnRHant (Table 2). However, the animals treated only with GnRHant (Group IV) had a mild increase in the counting of preantral and antral follicles, resulting in a significant increase of the sum of follicles (17.1 ± 1.22) compared to the control group (10.9 ± 0.70 follicles, p < 0.05, Table 2).

**Table 2:** Ovarian morphological parameters in rats following chemotherapy and pregnancy.

	Group I (placebo)	Group II (CPA)	Group III (GnRHant + CPA)	Group IV (GnRHant)
Primordial and primary follicles	3.4 ± 0.53	2.9 ± 0.38	3.8 ± 0.46	3.8 ± 0.52
Preantral follicles	3.0 ± 0.24	3.4 ± 0.54	3.9 ± 0.48	4.1 ± 0.39
Antral follicles	4.4 ± 0.34	5.0 ± 0.60	6.6 ± 0.53	6.4 ± 0.50
Corpora lutea	4.2 ± 0.40	3.3 ± 0.50	3.4 ± 0.40	4.1 ± 0.30
Total number of follicles	10.9 ± 0.70	11.6 ± 1.14	14.3 ± 1.26	17.1 ± 1.22*
Ovarian cross-sectional area (mm^2^)	5.45 ± 0.50	4.30 ± 0.56	4.56 ± 0.89	4.62 ± 0.66

Data are expressed as mean ± standard error. *p < 0.05 vs. Group I.

CPA, cyclophosphamide; GnRHant, gonadotropin-releasing hormone antagonist (cetrorelix).

## Discussion

Protection against the adverse effects of CPA on ovarian function is highly desirable given the number of women of reproductive age with disorders that are treated with CPA, and the improvement of survival rates in patients undergoing chemotherapy [21]. Evidence suggests that prepubertal patients are less sensitive to the adverse effects of chemotherapy [22]. Thus, drugs that are able to make the gonads quiescent and less sensitive to chemotherapy-induced cytotoxicity could protect fertility.

Delaying cancer treatment can pose serious risks to the patient. Therefore, a drug that promotes immediate ovarian suppression is preferable. Given the immediate mechanism of action of GnRH antagonists, their use would eliminate the 14-day waiting period required to suppress ovarian activity using GnRH agonists.

In this study, the mean litter size of rats treated with a GnRH antagonist plus CPA was greater than that in the group that received only CPA without GnRH antagonist co-treatment, suggesting that antagonists may protect reproductive function. This result may be due to the immediate interruption of gonadotropic stimulus on the ovaries, causing a reduction in mitosis within the gonad and a transient follicle growth arrest, which renders the follicles less susceptible to the aggression of chemotherapy [22,23]. However, these mechanisms deserver further investigation in order to demonstrate their precise role in ovarian chemoprotection, and also to test whether GnRHant has any direct effect on the ovaries.

Estrous cycles were not permanently suppressed by CPA, at least in the short term following the chemotherapy course. This result is similar to that reported by Ghosh *et al*. [24], who noted, nevertheless, an increase in the metaestrus and diestrus and a decrease in the proestrus and estrus. This was confirmed here by the lower number of rats showing estrus or proestrus after CPA treatment, compared to placebo. Moreover, the reversion of this abnormality by pre-treatment with GnRH antagonist is suggestive of a protective effect on ovarian endocrine function.

In the present study we have not observed substantial changes in ovarian morphology in any treatment groups, while previous studies have shown a decrease in the number of primordial follicles following chemotherapy [6,7,25]. A possible explanation for this difference could be because we evaluated ovarian morphology after a full term gestation, while others have extracted the ovaries soon after chemotherapy, without the interference of pregnancy. However, this study was designed to evaluate not only potential fertility, through ovarian morphology and estrous cycle, but actual fertility by assessing pregnancy and live births, which only allowed oophorectomy to be performed after gestation and delivery.

A significant decrease in ovarian cross-sectional area following CPA administration was not seen in our study. The most likely explanation for this is that the rats were submitted to a single course (three cycles) of chemotherapy, which is enough to cause fertility impairment, but may have been insufficient to change ovarian size. It is not clear whether several courses of CPA would decrease the ovarian cross-sectional area, or whether the follicles that were found in the specimens were viable; that is, whether they would be able to produce mature follicles and a subsequent pregnancy. Moreover, although the number of follicles and ovarian cross-sectional area were not altered by CPA treatment, the number of pups in the offspring was lower, indicating a deleterious effect on fertility. It is important to note that ovarian morphology does not necessarily reflect its endocrine or reproductive function. Therefore, the residual follicles that we observed after chemotherapy and pregnancy may not be all normally functional, as suggested by the fertility impairment of rats receiving only CPA.

The results of this study support the hypothesis about fertility protection by GnRH analogs in female rats undergoing chemotherapy with CPA. Several studies have highlighted this protection with the use of GnRH agonists. Most of the work are still experimental, but some clinical studies are now being conducted in this field. Although some cancer centers are already offering this option to protect ovarian function, both the American Society for Reproductive Medicine (ASRM) and the American Society of Clinical Oncology (ASCO) discourage this practice due to insufficient scientific evidence [26,27]. However, these same entities, based on the results found by animal studies, encourage the development of research in this area and recommend this as a promising possibility. The advantage of the use of GnRH antagonists instead of agonists would be their ability to promote a prompt gonadal suppression, allowing the immediate start of chemotherapy, which could be crucial in the treatment of cancer.

To the best of our knowledge, only two published studies have evaluated the use of cetrorelix for ovarian protection in animals subjected to chemotherapy with CPA. Both studies investigated only the number of primordial follicles in female mice treated with CPA, and none of them directly assess fertility. Meirow *et al*. [25] concluded that the use of cetrorelix attenuates the reduction in the number of primordial follicles caused by CPA, suggesting, therefore, ovarian protection with the use of cetrorelix. This protection was not found by Danforth *et al*. evaluating the primordial follicles in female mice subjected to a single dose of CPA and GnRH antagonist [28]. However, in the latter study the results should be interpreted more cautiously because the number of animals was small, the regimen of chemotherapy was a single dose and the only variable considered was the number of primordial follicles.

The safety of the use of cetrorelix in humans is already consolidated and this drug is widely used for ovarian blockade in patients submitted to assisted reproduction techniques. This proven safety can facilitate the development of clinical studies with the goal proposed here.

## Conclusion

The administration of GnRH antagonist cetrorelix had a protective effect on the fertility of rats undergoing chemotherapy with CPA.

## Competing interests

The authors declare that they have no competing interests.

## Authors' contributions

CNCDL conceived the study and design, carried out the bibliographic research, supervised data acquisition, analyzed the results and drafted the manuscript. FMR was the project co-supervisor, chose and coordinated the statistical analysis, data interpretation and manuscript revision. GNP participated in the animal experiment and acquisition of data. LCS participated in the animal experiment and acquisition of data. AFC was the project supervisor and developed the idea. All authors read and approve the final manuscript.
